# Success and Challenge When Returning to Clinical Practice: A Case Series in Anesthesiologist Re-Entry

**DOI:** 10.1155/2019/3531968

**Published:** 2019-12-20

**Authors:** Michael S. Green, Usama Iqbal, Christopher R. Hoffman, Parmis Green, Nielufar Varjavand

**Affiliations:** ^1^Department of Anesthesiology and Perioperative, Drexel University College of Medicine, Hahnemann University Hospital, Philadelphia, PA, USA; ^2^Department of Internal Medicine, Drexel University College of Medicine, Hahnemann University Hospital, Philadelphia, PA, USA

## Abstract

**Introduction:**

Anesthesiologists returning to clinical practice pose unique challenges for licensing and credentialing boards. Few institutions provide re-education. We describe the physician refresher/re-entry program at our College of Medicine.

**Methods:**

We launched the physician re-entry program in 2006. This individualized program re-educates physicians who left clinical practice for any reason and are seeking to return. We report results achieved for 12 anesthesiologists who successfully completed the course between August 2012 and February 2018.

**Results:**

Seven men and five women left their practices for various reasons, which included relocation, family or medical reasons, substance use, and burnout. None left practice for medical negligence. Range away from clinical activity was 0–10 years. Five had active licenses. Seven were US graduates and five were international. Nine of 12 achieved their goals. Of the 3 others, 1 did not pursue her goal, another did not obtain a residency, and the other just finished the program. Seven out of 9 (78%) achieved their goal within 1 year of course completion.

**Discussion:**

Despite our small sample size, our experience to successfully return inactive physicians to the workforce adds to the scant literature and experience in refreshing inactive physicians. Our trainees return to practice serving communities across the country and are now a pivotal part of the anesthesiology workforce. Thus, this program not only services individual physicians, but the whole community affected by their absence.

## 1. Introduction

Practicing physicians leave medicine for multiple reasons including illness, family responsibilities, career dissatisfaction or change, substance use, and early retirement [1, 2]. Returning to practice can be challenging for many reasons. State licensing boards and hospital credentialing processes have various re-entry policies, including demonstration of baseline knowledge, ongoing continuing medical education (CME), and completion of a formal refresher program. Some recruiters require re-entry re-education with a six-month clinical gap. The American Medical Association recommends that a physician absent from practice for 2 or more years participates in a formal program capable of assessing essential clinical competencies and refreshing relevant skills for workforce re-entry [3]. The American Board of Anesthesiology written policy on reattaining certification status dictates that consideration is an individualized, case-by-case basis. Registrants may be required to “undertake continuing medical education, complete additional training, and complete other activities deemed necessary” [4]. In addition to professional and institutional challenges, an anesthesiologist re-entering practice after a period of inactivity may face personal obstacles as well. Low self-confidence, limited resources in acquiring up-to-date knowledge, and a lack of professional network may limit success.

Few programs address the professional and educational needs of inactive physicians, especially in anesthesiology [5]. In general, programs evaluate the re-entering or remediating physician through assessment for competence using multisource examinations [6]. The American Society of Anesthesiology lists two re-entry programs that provide education for the inactive physician seeking return to practice in New York and Philadelphia. Both the aforementioned programs provide re-education during observation alongside other learners; both provide varying program duration based on clinical time away. A new program in Texas (KSTAR/UTMB) has just started. We report on the refresher program at Drexel College of Medicine that facilitates anesthesiologists' return to clinical practice.

## 2. Methods

### 2.1. Program Overview

In 1968, the Medical College of Pennsylvania was among the first universities to initiate a re-entry program for nonpracticing physicians returning to clinical practice [7, 8]. Successful with over 400 trainees, the program temporarily ceased in 1993 when the hospital closed. In 2006, it was reinstituted and redesigned based on six core ACGME competencies to parallel current medical education, with adult learning principles. While the program maintains a basic structure of formative assessment, education, and then reassessment in a university-teaching environment with individual and group learning and a dedicated preceptor, the details are individualized (for the adult learner) based on the physicians' goals. A steering committee was convened with assistant deans of education, including CME and assessment, to guide the redesign of the program. The committee meets regularly in an advisory role, including review of applicants. While the director oversees the entire Medicine Physician Refresher/Re-entry Program broadly, each department has dedicated preceptors to provide specialty-specific one-to-one education and mentoring. At the same time, the trainee learns with the faculty in their specialty-specific department. The program began with internal medicine and pediatrics; because of increasing demand, we developed the anesthesiology track in 2012. In addition to a clinical and didactic refresher, the individualized, structured program provides administrative support (through the continuing medical education (CME) department), career counseling, and postprogram follow-up to address requests from credentialing groups. Alumni, randomly and on their own volition, often stay in touch for years to share their accomplishments.

Upon initial contact, the program director and returning anesthesiologist discuss relevant career background, anticipated future practice scope, reasons for leaving and returning, organizational requirements, and specific goals or expectations. Then, if the physician desires, the program director communicates with referring groups to further identify parameters for the physician's individualized re-education. All initial application review is by the director, steering committee, and then specialty-specific preceptor. The director and preceptor further conduct phone interviews with trainees to assure that goals can be met. Accepted trainees are enrolled in 6-week blocks (originally selected to match the medical school block duration) for 6, 12, or more weeks depending upon the individualized goals and recommendations. To optimize focused attention, in the anesthesiology department, one re-entry physician is accepted at any one time.

The distinct outcome of this re-entry program is the refresher and update of skills are necessary to practice anesthesia as the American Board of Anesthesiology would deem fit. This includes sufficient knowledge base, application of knowledge to develop sound plan, and reinsertion into CME cycle requisites. Re-entering physicians, each with their own specific obstacles and needs seek a refresher; we ask them to identify their primary goals. Ascertaining said goals helps to further personalize the program, address each learner's individual needs, and fine-tune interactions with preceptors. We believe that going beyond the textbook and organizational needs to address the varied backgrounds of participants is crucial to fostering a constructive, successful environment.

The program director, physician trainee, preceptor, and program coordinator develop an individualized curriculum. Several components of the program are mandatory; however, within the mandatory components, the individual cases and days can be adapted based on the physician's career goals. This process is informed by an initial needs assessment, during week 1, over a 2-day period, including Post Licensure Assessment System (PLAS) multiple-choice examinations, oral discussions of clinical scenarios, three standardized patient evaluations and their documentations. The information from the needs assessment is reviewed with the student over a two-day long feedback didactic session in a group setting. Concepts are reinforced, or new concepts introduces, as trainees learn from one another as well as the faculty. This allows trainees to contribute to each other's experiences and expand learning from everyone's topics and issues. The re-entry physician also completes a clinical interest survey to communicate their focus of interest for program scheduling. While trainees all follow the overall structure of the preceptorship, there is flexibility based on needs and goals within each component. For instance, all trainees receive formative feedback on their standardized patients' evaluations. If a trainee needs more practice with communication skills versus medical content, then cases for practice are selected to meet their individual needs. Another example is in the self-selection of the anesthesia cases among all operating rooms, based on the physicians' individual goals. Trainees keep a daily patient/case log to assure a wide range of clinical exposures that meet their specific future work goals. Trainees also write pre/postop notes for practice, on encountered patients in a word document (not in the patient's actual chart), which they review weekly with their preceptor for summative feedback. Preceptors together observe the trainee's recorded standardized patient scenarios (done as pretest, practice, and posttest) using a communication skill and medical content checklist to identify strengths and potentials for improvement. Updating computer, presentation, and peer teaching skills are other aspects of training.

### 2.2. Re-Entry Program Components

#### 2.2.1. Clinical Setting

Similar to the program at Mt. Sinai, this program does not provide a hands-on experience for inactive returning anesthesiologists [9]. Our program seeks to gradually immerse the physician back into a clinical setting through didactics, discussions, observation in the OR alongside other learners for 8 hours/day, and standardized patient testing with feedback. These are all controlled exposures to clinical scenarios in an environment with the anesthesia team present, accounting for the limitation that inactive physicians do not often have an active license and thus are not able to obtain malpractice insurance [10]. Furthermore, similar to Mt. Sinai [9], we have found that pursuing relicensure, hospital credentialing, and current malpractice tremendously adds to the cost and time to start a re-entry program for physicians, rendering the re-entry program not feasible.

In a tertiary academic center in an urban setting, physicians are exposed to an all-encompassing variety of real patient cases highly beneficial to a re-entering physician, who are seeking to update their skills and regain their confidence. Re-entering physicians choose from a myriad of available cases to suit their individual needs based on their self-assessed and practical future goals, similar to adult learning models. Candidates do not perform hands-on procedures but have direct interaction with the patients in the perioperative setting. Day-to-day discussions of comorbidity and subsequent anesthetic plans are reinforced by preceptors at the bedside. Re-entry physicians follow these patients into the operating room and witness their entire induction, maintenance, and emergence. They witness the assessment and recovery process in the postoperative care unit. Throughout this process, they are expected to actively interact, question, and learn from the perioperative team. We have learned that after applying didactic learning to such experiences, physicians are confident and academically up-to-date to enter a clinical setting where their employers often then provide an initial short supervised period.

#### 2.2.2. Standardized Patient Evaluations

Two to three times during each 6-week period (pretest, posttest, and practice based on the individual need), physicians evaluate standardized patients for formative and summative feedback. Cases have been written by Drexel physicians (subspecialist and primary care physician-educators, and reviewed by a committee of peers); there is access to a large variety of scenarios, both for communication and clinical content. The cases and checklists are standardized, identical to ones used for Drexel medical students, residents, and physician re-entry trainees. The level of the re-entry anesthesiologist is different from that of the other learners, but the content still proves useful. After the evaluations, as a group in an interactive setting where all contribute, learners, and faculty review the recorded evaluations, provide feedback, and are scored based on standardized checklists. The checklists measure interviewing skills as well as content medical knowledge. The content medical knowledge lists questions a physician should ask to elaborate a focused history of the specific chief complaint and elicit/narrow its differential diagnoses. The interviewing skills measure data gathering, interpersonal, information giving, and organizational skills as well as standardized patient satisfaction. As such, physicians first practice, learn, and improve in a safe and controlled environment.

#### 2.2.3. Web-Based Modules

To assure reentering physicians' re-education parallels current up-to-date medical education, which may have been different from the physician's early medical education, we incorporate technology as a learning tool. In addition to in-person didactics and discussions in anesthesia conferences, the curriculum includes web-based interactive programs for focused learning and assessment on medical knowledge, communication skills, and clinical reasoning. Three distinct sources provide these online annotated video and virtual patients (DxR https://dxrgroup.com/healthcare-education-products/dxr-clinician/, Doc.comhttps://doc.com/, and Aquafer https://www.aquifer.org/). Each program has its own learning objectives. Participants are expected to complete 1-2 modules (self-selected based on goals) per week in each program and encouraged to redo modules as reinforcement, for practice in a virtual and safe setting. Didactic learning is assessed via multiple-choice or short-answer exam questions with feedback. Trainees report that these resources are invaluable for their learning and often voluntarily complete more modules than their weekly assignments.

#### 2.2.4. Medical Documentation

Trainees write medical notes regarding patients they had observed in the electronic health record (EHR) format to reinforce documentation skills, which they review through case logs with preceptor weekly. The templates are from the EHR, copied as pdf file where trainees may write their notes, but they are neither online nor part of the patient's permanent records; the documentations provide room for practice and learning. Trainees could participate in further EHR training with administrators, based on their individual needs.

#### 2.2.5. Preceptorship

Trainees participate in grand rounds, clinical cases, and conferences. Trainees learn within a multidisciplinary team with the entire anesthesiology staff (faculty, nurses, and residents) and self-select to work closely with faculty who share their interest. Each physician regularly meets one-on-one with a designated anesthesiology mentor for two hours for further individualized learning. These meetings include oral presentations, discussion of daily OR cases, review of notes, and summative performance evaluation.

#### 2.2.6. Simulations

Trainees participate in simulated scenarios in a multidisciplinary setting alongside nurses and other healthcare providers using the same simulations used to teach anesthesiology residents. These sessions include trained individuals as simulated patients to create realistic patient encounters, including but not limited too medical history obtain, consenting, conflict resolution, and ultrasound training. Robotic models that display changeable vital signs, can be “administered” medication, and be subjected to procedures which mimic acute scenarios. Sessions take place in the simulation center and are designed to benefit all learners involved; anesthesia trainees gain personal and interdisciplinary experience in this fashion. Learners are able to participate as the primary clinical provider and observe others. Each trainee has at least one simulation session during the re-entry period. These sessions are followed by debriefing sessions where the scenarios are discussed in detail for learning opportunities.

#### 2.2.7. Computer Searches and Critical Appraisal Training

During the orientation week, students meet with the science librarian for information technology skill sessions to learn evidence-based medicine research skills for continued self-improvement after the program. Trainees are expected to use these skills during their presentations; they are required to make one formal presentation on any self-chosen topic to their peers as well as volunteer for informal self-selected clinical presentations.

#### 2.2.8. Advocacy and Support

Most re-entry physicians confront emotional, personal, career, and financial barriers when returning to practice. The continuing medical education department provides hours of career and emotional support before, during, and after the program. We advocate on trainees' behalf by writing letters, brainstorming career options, providing networking and employment introductions, and speaking to committees/employers.

#### 2.2.9. Performance Evaluation

Weekly evaluations monitor achieved learning goals. We regularly seek trainees' written and verbal feedback, review gaps, and adjust schedules. Faculty provides summative feedback weekly on trainees' clinical knowledge, communication skills, professionalism, history taking and documentation skills, and formative feedback using evaluation forms also used for medical students. Since all faculties participate in teaching medical students and residents, they use these evaluation forms regularly. Each trainee receives a minimum of 1 evaluation per week of the course. Upon completion, each trainee receives a detailed evaluation letter and certificate explaining their accomplishments and assessments; individual faculty feedback is listed, as well. The letters do not endorse competency for practice; rather they report on physicians' accomplishments.

## 3. Results

Results reflect 6-year data from anesthesiology department re-entry physician trainees, from August 2012 to February 2018. Twelve physicians began and completed the anesthesiology re-entry program. Trainee demographics are in Table 1. Each application was reviewed; none were rejected. Seven out of twelve were US medical graduates, five were international medical graduates, four were board-certified, and five had active licenses. Range of clinical inactivity was 0–10 years. Those who had no gaps had been immediately practicing in a global health setting but relocated to the US (one while in anesthesia residency in the United Kingdom, the other while practicing in South Africa). Even though they did not have a gap in their careers, each career setting provides its own needs and skills, and international physicians must complete US residencies. These physicians sought to update their skills before seeking residencies. Only one was practicing as an anesthesiologist; six were not working; the others worked in various capacities: insurance, high school teacher, postdoctoral student, and physician in another capacity (emergency, general practitioner, and chronic pain). Reasons for leaving and returning to clinical practice were: 1 relocation, 4 substance abuses, 1 family issue, 1 medical disability, 1 burnout, and 1 other. Those with substance abuse history had successfully completed programs with the Physicians' Health Program. None had left practice for medical negligence.

Each physician noted their specific goals after program completion (Table 2). Nine of twelve trainees achieved their individual goal (75%); 7 (78%) achieved their goal within 1 year of course completion. Six pursued residency training. Five sought employment. One sought license renewal prior to seeking employment. Of these six who sought employment and five (82%) immediately began work. Four (67%) of six seeking residencies obtained spots (one of the two has not tried yet). Three did not achieve their goals; two were seeking residencies (one had left her residency to raise a family, another was IMG moving to US); and one just finished the program.

## 4. Discussion

Returning physicians bring unique backgrounds, needs, skills, and knowledge from their nonclinical experiences. Inactive physicians face difficulty returning to work for various reasons. Increasingly, they are asked to gain re-education. A re-entry program should provide flexible individualized re-education to meet physicians' varying needs. Assessment is important for ongoing improvement, and a re-entry program is an effective way by which physicians can seek assessment and improve their professional skills [2, 10, 11]. This program meets the AMA Guiding Principles [3]; physicians seek the program from all geographic regions and note its national and international reputation. Although twelve physicians of differing backgrounds do not represent all returning physicians' needs, our approach and practical experience alongside work done by other programs can provide an initial guide for the growing field of anesthesiology re-entry [9, 11–14].

We seek physician feedback for constant improvement; thus, we have learned important lessons. A multidisciplinary team of faculty, resident, and nursing support is critical to trainee re-education. Clinician-educators are best suited for re-entry physicians, as they are at the forefront of education. Re-education is best among a community of learners alongside fellows, residents, and students. The university provides undergraduate and graduate medical education; anesthesiology re-entry physicians learn alongside a variety of learners. This variety of knowledge distribution strengthens clinical skills and promotes the learning process. Interactions within this structure help to boost confidence and teaching skills. The re-entering anesthesiologist contributes to the learning of others by bringing nonclinical experiences.

We explain choices available to trainees and obstacles they may face during or after the course. After hours of career and personal counseling, some may opt not to pursue re-entry, as with one of the trainees. Randomly, some trainees maintain close contact with us years later, reaching out for news or even guidance. We have expanded our career advisory roles, as trainees return for networking and advice long after program completion. We have an alumni association, where current trainees communicate (online or in person) with alumni for questions and support. Despite challenges of re-entry, participants are eager to regain proficiency and as evident in our program, the majority has succeeded in achieving their anticipated goals.

The re-entry program has been a part of the College of Medicine since 1968, yet challenges still persist. While the re-entering physicians may learn in one department, resource utilization in other departments (CME, library sciences, and standardized patient program) requires timely coordination. While an academic setting is ideal for education and patient scenario variety, at the same time there are many learners (students, residents, and fellows) which necessitate clever and planned space coordination. Costs are significant in time and personnel due to the need for dedicated staff and faculty [11]. Two program coordinators help organize details: one is full-time in CME who coordinates all re-entry physicians' schedules with their individual departments' corresponding coordinator. This person has other duties too.

Follow-up with re-entry physicians to ascertain goal completion in short term is achieved via direct contact with participants. These individuals often keep informal contact with the program to share their success and positivity. As years progress, it becomes difficult to maintain contact and ensure long-term achievement. Further program improvement includes developing dedicated means to contact destination sites to ascertain trainee status and provide long-term support.

This paper reflects 12 physicians' experiences; there is clear need for continued program evaluation. Our general re-entry program originally began in 1968; the anesthesiology track was developed in 2012. While this physician cohort, with representation from all regions and backgrounds, has had a 75% success in reaching their individual goals, we reflect short-term data upon program completion. Although our results are encouraging, there are more questions that need further investigation. Of specific note, areas of interest for future study could include which learning activities were of most use, if coaching support was beneficial, or if supervision upon reinstitution to practice helped support reintegration. For instance, it would be interesting to note if re-entering physicians more readily leave medicine again; or if they left for medical reasons, is relapse common; or do they enter advocacy fields to help colleagues maintain practice. Even as such, this anesthesiology physician re-entry program can provide a useful service to the community.

## 5. Lessons for Practice

- A multidisciplinary team of faculty, resident, and nursing support is critical to trainee re-education.
- Clinician-educators are best suited for re-entry physicians, as they are at the forefront of education, and this is best accomplished among a community of learners alongside fellows, residents, and students.
- The re-entering anesthesiologist contributes to the multidisciplinary variety of knowledge and strengthens clinical skill that accelerates the learning process along with a boost of confidence.

## Figures and Tables

**Table 1:** Anesthesia trainee demographics.

Anesthesia trainee demographics	
Gender	*n*
Male	7
Female	5

Location	*n*
US graduate	7
International graduate	5

Board certification	*n*
Maintained	4
Not maintained	8

Licensure	*n*
Active	5
Not active	7

Reason for leaving	*n*
Relocation	4
Substance abuse	4
Family issue	1
Medical disability	1
Burnout	1
Other	1

**Table 2:** Main goals and outcomes for trainees.

Main goals and outcomes for trainees who completed the physician refresher/re-entry course			
Goal	*n*	Outcome	*n*
License	1	License successfully reinstated	1
Employment	5	Refreshed and practicing	4
Residency	6	Residency training	4
		(i) Decided not to seek residency	1
		(ii) Just completed the program	1

## Data Availability

The data used in this case series are presented in the article. Data not presented in the manuscript are not to protect subject privacy.
